# An electrophoretic mobility shift assay using the protein isolated from host plants

**DOI:** 10.1186/s13007-024-01201-7

**Published:** 2024-05-12

**Authors:** Zihang He, Zhibo Wang, Zhangguo Lu, Caiqiu Gao, Yucheng Wang

**Affiliations:** State Key Laboratory of Tree Genetics and Breeding, Northeast Forestry University, Harbin, 150040 China

**Keywords:** EMSA, Fluorescence, Cyanine 3, DNA-protein interaction

## Abstract

**Background:**

The electrophoretic mobility shift assay (EMSA) is a common technology to detect DNA-protein interactions. However, in most cases, the protein used in EMSA is obtained via prokaryotic expression, and rarely from plants. At the same time, the proteins expressed from prokaryotic systems usually cannot fold naturally and have no post translationally modification, which may affect the binding of proteins to DNA.

**Results:**

Here, we develop a technique to quickly isolate proteins of interest from host plants and then analyze them using fluorescent EMSA. This technology system is called: protein from plants fluorescent EMSA method (PPF-EMSA). In PPF-EMSA, a special transient transformation method is employed to transiently deliver genes into the plant, enabling efficient synthesis the encoded proteins. Then, the target protein is isolated using immunoprecipitation, and the DNA probes were labeled with cyanine 3 (Cy3). Both fluorescent EMSA and super-shift fluorescent EMSA can be performed using the proteins from plants. Three kinds of plants, *Betula platyphylla*, *Populus. davidiana×P. bolleana* and *Arabidopsis thaliana*, are used in this study. The proteins isolated from plants are in a natural state, can fold naturally and are posttranslationally modified, enabling true binding to their cognate DNAs.

**Conclusion:**

As transient transformation can be performed quickly and not depended on whether stable transformation is available or not, we believe this method will have a wide application, enabling isolation of proteins from host plant conveniently.

**Supplementary Information:**

The online version contains supplementary material available at 10.1186/s13007-024-01201-7.

## Background

Gene expression is regulated, in part, by transcription factors (TFs) that bind to specific sequence motifs in genomic DNA. TFs cooperate with the basal machinery to upregulate or downregulate gene transcription [1]. DNA-binding TFs target distinct DNA sequences at gene regulatory regions, thus ensuring specificity in the assembly of the transcription machinery [2, 3]. Protein-DNA interactions play a significant role in many biological processes, including gene expression, transcription, DNA replication, DNA repair, and chromosomal DNA packaging [4–8].

The electrophoretic mobility shift assay (EMSA) or band shift assay, which is a powerful molecular biological method to detect proteins that bind to specific DNA oligonucleotides, was established in 1981 by Fried and Crothers [9]. The basis of EMSA is the reduction in the distance travelled through a nondenaturing gel by a DNA or RNA molecule when it is bound to protein(s) [10, 11]. Several antibody-based procedures can be used to identify proteins in DNA-protein complexes: (i) Immunoprecipitation (IP) of ultraviolet crosslinked DNA-protein products; (ii) immunoblotting of an EMSA gel; and (iii) band retardation by antibodies added to the protein-DNA binding reaction (super-shift assay) [12]. EMSA can be used with DNA fragments of almost any length to determine the relative binding affinities of interactions, and is simple and cost-effective [13, 14]. However, EMSA suffers from several limitations. First, the total time required to obtain the results is 4–10 h and it can take even longer to identify low-abundance interactions [14]. Second, EMSA has a limited dynamic range because it is mostly applicable to high affinity interactions [15]. Third, the protein-DNA complex might dissociate during the electrophoresis step [14]. Several parameters might influence the DNAprotein interaction, which have been reported previously: including binding buffer and temperature, duration of complex formation, the gel concentration and composition, and the running temperature. Subsequently, several different EMSA variants have been developed, such as Reverse EMSA [16], Topoisomer EMSA [17], Cryogenic EMSA [18], and EMSA followed by mass spectrometry [19]. The original method and all its modifications are based on the capacity of labeled nucleic acid oligonucleotides that are bound to proteins to move slower through a polyacrylamide gel matrix than the unbound labeled oligonucleotides [14]. However, the classic chemiluminescent EMSA is not only complex, but also has low sensitivity. In addition, most EMSA methods use protein obtained by prokaryotic expression, and rarely use the protein from the host organism.

Radiolabeling of DNA using ^32^P has been the predominant method of detection in EMSA, which is sensitive and beneficial; however, there are health and safety risks associated with the use of radioactivity. Thus, alternative DNA labeling methods with comparable sensitivity have been employed, including conjugation of DNA with digoxigenin (DIG) [20] or biotin [21]. However, these methods involve extra processes, such as membrane transfer and chemiluminescent detection. SYBR green staining of the polyacrylamide gel electrophoresis (PAGE) gel requires post-electrophoresis gel staining and a fluorescent scanner [22]; however, this means that the resolved PAGE gel can be assayed only once. In contrast, fluorescent EMSA uses DNA probes labeled with fluorophores, which can be directly detected in the gel, and is more sensitive than chemiluminescent EMSA. In addition, the DNAprotein interactions can be visualized in real time during electrophoresis, which would reduce cost and time significantly. The fluorescent dyes used to label probes include cyanine 5 (Cy5) [23, 24], cyanine 3 (Cy3) [23, 24], hexachlorofluorescein (HEX) [25], IRDye-800 or DY-781 [26] and infrared fluorescent dyes [22, 24, 27, 28].

In the present study, we developed a method to isolate proteins from plants, which was combined with fluorescent EMSA to detect host plant protein-DNA interactions. The protein from the host plants is in its natural state and has post-translational modifications (PTMs), which can affect the binding of protein to DNA; therefore, the protein from host plants might reflect the true binding of the protein to DNA. This EMSA system may have wide application in studies of DNA-protein interactions.

## Materials and methods

### Plant materials

*Betula platyphylla* (birch) and *Populus. davidiana×P. bolleana* (Shanxin poplar) plantlets were grown in growth medium (Woody Plant Medium [2.41 g l^− 1^] + 1 mg l^− 1^ 6-Benzylaminopurine, pH 6.0) in a tissue culture room under the following culture conditions: An 8/16 h cycle of darkness/light, light intensity of 400 µM m^− 2^ s^− 1^, relative humidity of 70%, and a temperature of 24 °C. Seeds of transgenic *Arabidopsis thaliana* (Arabidopsis) lines were seeded into pots containing a mixture of perlite/soil (v/v = 2:1) in a greenhouse under the condition of 16-h light/8-h darkness photocycle and 70-75% relative humidity at 22 °C.

### Protein prokaryotic expression and purification

The coding sequence (CDS) of the TFs BpERF3 (Ethylene Response Factor 3, GenBank number: KM980047) from birch and PdbWRKY46 (a WRKY TF, GenBank number: PP035547) from Shanxin poplar, were fused separately with a plasmid encoding the maltose-binding protein (MBP) to generate pMAL-C5x-BpERF3 or pMALC5x-PdbWRKY46 vectors, which were transformed into *Escherichia coli* (*E. coli*) 2523. All the primers were shown as the Supplementary Table 1. The transformants were grown in Luria-Bertani (LB) liquid medium containing ampicillin at 37 °C until the optical density at 600 nm (OD_600_) reached 0.5 and then 1 mM Isopropyl βd-1thiogalactopyranoside was added and incubated for 2 h to induce protein expression. Cells were collected by centrifugation, and dissolved using chemotaxis buffer (20 mM Tris-HCl pH 7.4, 0.2 M NaCl, 1 mM EDTA, 1 mM dithiothreitol [DTT]). The cells were sonicated at 20% power output (900 W max power output) for 10 min with a cycle of sonication for 5 s and pausing for 10 s (Ultrasonic Cell Crusher JY92-IIDN, Scientz, Ningbo, China). After sonication, the cells were centrifuged at 12,000 × *g* for 10 min, and the supernatant was transferred into a new tube. A portion of the supernatant was taken for sodium dodecyl sulfate (SDS)-PAGE analysis. Amylose resin was used to affinity purify BpERF3MBP and PdbWRKY46-MBP. After washing, the BpERF3-MBP or PdbWRKY46-MBP proteins were eluted by displacement with 10 mM maltose, and was stored at -80 °C until use.

### Vector construction and DNA labeling

The CDS of *BpERF3* or *PdbWRKY46* were fused with a FLAG epitope (5’-GATTACAAGGACGACGATGACAAG-3’) and cloned into vector pCambia1307 separately for transient plant overexpression (termed p1307-*BpERF3*-Flag and p1307-*PdbWRKY*-Flag). For RUBY detection, 35S: RUBY harbored in pHDE vector was used in plant transient transformation. For BpERF3 probe labeling, a truncated promoter of the *WRKY28* (299 bp) that can be bound by BpERF3 [29] was used as the probe sequence, and the primers to amplify this region were designed and labeled with biotin or Cy3 at the 5’ end, respectively. PCR was performed using the *WRKY28* promoter as a template to label the PCR product with biotin or Cy3 as the probe, and the PCR product was purified by Tris-phenol and chloroform extraction. After extraction, a 3-fold volume of ethanol and glycogen was added, and the mixture was centrifuged at 12,000 × *g* for 10 min to precipitate the probe. For PdbWRKY46 probe labeling, a paired single strand DNA containing three tandem copies of the W-box was synthesized and labeled with Cy3 at the 5’ end (Sangon, Shanghai, China). The labeled probes were mixed in a buffer (10 mM Tris-HCl, 2.5 mM MgCl_2_, 50 mM KCl, pH 9.0), and annealed into a double stranded DNA as the probe by incubating at 98°C for 2 min, then leave it at room temperature for 30 min. Usually, the probes less than 90 bp can be synthesized directly in a company, and the probes more than 90 bp can be synthesized the primers labeled with Cy3 at the 5’ end using PCR. All the primers were shown as the Supplementary Tables 2 and S3.

### Chemiluminescent EMSA

To perform chemiluminescent EMSA, the purified protein was incubated with the DNA probes labeled with biotin at the 5’ end, and the unlabeled probes were added as competitors. The EMSA assay was performed using a chemiluminescence EMSA kit (Beyotime, Jiangsu, China). In brief, all the samples were electrophoresed at 150 V through non-denaturing polyacrylamide gels (PAGE) at different concentrations (1×TBE containing 80% glycerol, 10% Ammonium Per Sulfate [Aps], 30% Acr-Bis [29:1] and 0.1% [v/v] TEMED), and were then transferred to a nylon membrane via electroblotting. After ultraviolet crosslinking on membrane that is irradiated for 3–10 min at a distance of about 5–10 centimeters from the membrane using portable UV detector (EUV002) (Beyotime, China), the bound complexes were conjugated with Streptavidin-horseradish peroxidase (HRP), and visualized using enhanced chemiluminescence in the Tanon 5200 CCD system (Tanon, Shanghai, China). All the primers were shown as the Supplementary Table 3.

### Plant genetic transient transformation

The detailed procedures for transient transformation were as the follows: (1) A single clone of *Agrobacterium tumefaciens* (*A. tumefaciens*) strain EHA105 was grown in 5 ml of LB medium at 90 rpm at 28 °C. After the cells reached an OD_600_ of 0.8, 0.5 ml of the culture was added to 25 ml of fresh LB and incubated at 28 °C with 90 rpm shaking until the cell density reached an OD_600_ of 0.7. (2) The cells were harvested by centrifugation at 3,000 rpm, and adjusted to an OD_600_ of 0.7 using transformation buffer (2 mM MES-KOH pH 5.8, 40 mM CaCl_2_, 120 µM acetosyringone, 2% [w/v] sucrose, 270 mM mannitol, 20 µM 5-Azacytidine, 200 mg l^− 1^ DTT). (3) Whole plantlets (birch or poplar) were soaked in transformation buffer containing *A. tumefaciens* at 25 °C with 90 rpm for 1 h, then added with the same volume of fresh transformation solution (without *A. tumefaciens*) and incubated at 25 °C with 90 rpm for 1.5 h. (4) The plantlets were then washed twice with fresh transformation buffer (without *A. tumefaciens*) immediately to remove excess *A. tumefaciens* cells. (5) After washing, the plantlets were planted vertically on 1/2 Murashige and Skoog agar medium (1% [w/v] sucrose, 150 µM acetosyringone, 200 mg l^− 1^ DTT, pH 5.8) in bottles under at 24 °C with an 8/16 h of dark/light cycle. The transiently transformed plants can be used after transformation for 48–72 h.

### Western blotting and quantitative real-time reverse transcription PCR (qRT-PCR)

Total proteins were isolated using a Plant Total Protein Extraction Kit (PE0230-1KT) (SigmaAldrich, St. Louis, MO, USA), separated using SDS-PAGE, and then were transferred to a polyvinylidene fluoride (PVDF) membrane (Immobilon Millipore, Billerica, MA, USA) using a semi-dry blotting system. The membrane was blocked using Superblock (Perbio Science GmbH, Heidelberg, Germany) over night, and incubated with anti-Flag antibodies (Abmart, Shanghai, China) for 6 h. After washing five times, the membrane was incubated with HRP-conjugated antimouse antibodies for 2 h. After washing, the membrane was added with CDP-Star solution (Invitrogen GmbH, Karlsruhe, Germany) for 5 to 10 min. The signals were visualized using Pierce film (Perbio Science GmbH) and the Tanon 5200 CCD system.

Total RNA was isolated from the transiently transformed birch using Bioteke reagents (Beijing, China), reverse transcribed into cDNA using a PrimeScript reagent kit (Takara, Dalian, China), and diluted into 100 µl with ultrapure water as the qPCR template. The qPCR reactions were conducted on a qTOWER2.0 instrument (Analytical Jena, Jena, Germany). The reaction system for the qPCR step of the qRTPCR protocol included 10 µl of SYBR Green real-time PCR master mix (Takara), 0.5 µM of each forward and reverse primer, and 1 µl of cDNA template. The genes for *Tubulin* from birch and *Actin* from Shanxin poplar were respectively used as internal control to normalize the quantity of cDNA in each reaction. The thermal profiles were as follows: 94 °C for 30 s, followed by 40 cycles of 94 °C for 10 s, 59 °C for 30 s, and 72 °C for 40 s. Three independent biological replicates were used, and the relative expression levels were calculated using the 2^−ΔΔCt^ method [30]. See the Supplementary Table 4 for the primers.

### Protein immunoprecipitation (IP)

Transiently transformed plants (10–12 g) were used for protein IP. The detailed procedures were as follows: **Nuclei purification**: (1) The plants were ground into fine powder under liquid nitrogen, and the powder was suspended in 40 ml of buffer 1 (20 mM Tris-HCl pH 8.0, 1.5 mM MgCl_2_, 10 mM KCl, 5 mM DTT, 250 mM sucrose, proteinase inhibitors 1 µg ml^− 1^ each, 1 mM phenylmethylsulfonyl fluoride [PMSF]). After shaking at 120 rpm for 6 min, the solution was filtered through three layers of Miracloth (Calbiochem, San Diego, CA, USA). (2) The filtered solution was centrifuged at 300 × *g* for 10 min to precipitate the nuclei, and the nuclei were suspended in buffer 2 (20 mM Tris-HCl pH 8.0, 10 mM KCl, 1.5 mM MgCl_2_, 250 mM sucrose, 5 mM DTT, 1 mM PMSF, proteinase inhibitors 1 µg ml^− 1^ each, 0.1% [v/v] Triton x-100). The nuclei solution was centrifuged again at 300 × *g* for 10 min at 4 °C. **Nuclear protein extraction**: (3) The nuclei precipitate was suspended in 1 ml of buffer 3 (20 mM Tris-HCl pH 8.0, 420 mM KCl, 2 mM EDTA-Na_2_, 25% [v/v] glycerol, 1× phosphatase inhibitor cocktail, proteinase inhibitor cocktail 1 µg ml^− 1^ each, 1 mM PMSF), and then sonicated with 90 W power output for 2 min with the cycle of sonication for 2 s and pausing for 8 s. The sonicated solution was centrifuged at 12,000 × *g* for 3 min at 4 °C, and the supernatant was transferred to a new tube. **Concentration of nuclear proteins**: (4) 2 ml of buffer 4 (20 mM Tris-HCl pH 8.0, 1× phosphatase inhibitor cocktail, proteinase inhibitor cocktail 1 µg ml^− 1^ each, 1 mM PMSF), was added to the supernatant with and mixed well. (5) The solution was concentrated to 300 µl using an ultrafiltration column (Millipore, USA) with a cutoff of one-third of the molecular weight of the target protein; 2.7 ml of buffer 4 was added and the solution was filtered to 300 µl using an ultrafiltration column (30-kDa this study used). Then, 2.7 ml of buffer 5 (20 mM Tris-HCl pH 8.0, 165 mM NaCl, 1 mM EDTA, 7% [w/v] sucrose, 1 mM PMSF, proteinase inhibitors cocktail 1 µg ml^− 1^ each) was added and the solution was filtered to 300 µl using a 30-kDa ultrafiltration column. **IP using Flag magnetic beads**: (6) The solution of concentrated nuclear proteins was added with 300 µl of preprocessed Flag magnetic beads and incubated at room temperature for 2 h. A magnetic rack was used to separate the beads. The beads were washed using IP buffer (20 mM Tris-HCl pH 8.0, 150 mM NaCl, 1 mM EDTA) three times. **Elution**: (7) 200 µl of 3× Flag peptide solution (250 µg ml^− 1^) was added and incubated for 5 h at room temperature. The eluted products were filtered through an ultrafiltration column to a final volume of 50 µl.

### Fluorescent EMSA

**Probe labeling**: (1) Direct biosynthesis: when the probe was less than 100 bp, it was synthesized and directly fluorescently labeled (Cy3/Cy5 labeled) by a biotechnological company. (2) PCR labeling: The primers were synthesized and fluorescently labeled (Cy3/Cy5 labeled). PCR was performed using the fluorescence labeled primers, and the PCR product was purified by gel recovery. **DNA-protein interaction**: One microliter of the Cy3-labeled DNA (1 mM) and 7 μl of the protein were added to binding buffer (100 mM HEPES pH 7.5, 250 mM KCl, 10 mM MgCl_2_, 12.5 mM spermidine, 5% Ficoll400, 0.1 mM Zn(Ac)_2_, 2.5 mM DTT, 0.5 µg ml^− 1^ BSA) with total volume of 10 µl, and mixed well. The mixture was incubated at room temperature for 60 min. All the primers were shown as the Supplementary Table 3. **PAGE electrophoresis**: The DNA-protein mixture (10 µl) was loaded into a non-denaturing PAGE gel (6–7%, 1×TBE containing 80% glycerol, 10% Aps, 30% Acr-Bis [29:1] and 0.1% [v/v] TEMED) with clear protein loading buffer (45 mM Tris-Boric acid, 1 mM EDTA, 10% glycerol, pH 8.0). Electrophoresis was conducted on a Bio-Rad Protean III mini-gel (Bio-Rad, Hercules, CA, USA) with the voltage of 100 V, and running for 45 min. **Fluorescent visualization**: The gel was visualized with the Tanon 5200 CCD system. Super shift experiment was performed to validate specific interaction between DNA and protein by displaying two retarded bands using additionally anti-Flag antibody.

### Protein phosphorylation

Phosphorylated proteins were analyzed using immunoblotting detected with Phos-tag Biotin BTL-105 (WAKO Chemicals, Richmond, VA, USA). The proteins were separated with SDS PAGE, and phosphorylated proteins can bind specifically to Phos-tag Biotin that can be detected by the reaction between horseradish HRP and the ECL substrate as ECL signals.

### Statistics and reproducibility

Statistical analysis was performed using SPSS 16.0 software (IBM Corp., Armonk, NY, USA). The experiment was performed three times with similar results. Student’s t test and multiple comparisons (least significant difference [LSD]) were used to data comparison. Differences were considered significant if *p* < 0.05. In the figures, * and ** indicate *p* < 0.05 and *p* < 0.01, respectively.

## Results

### Transient transformation analysis

**Fig. 1:**
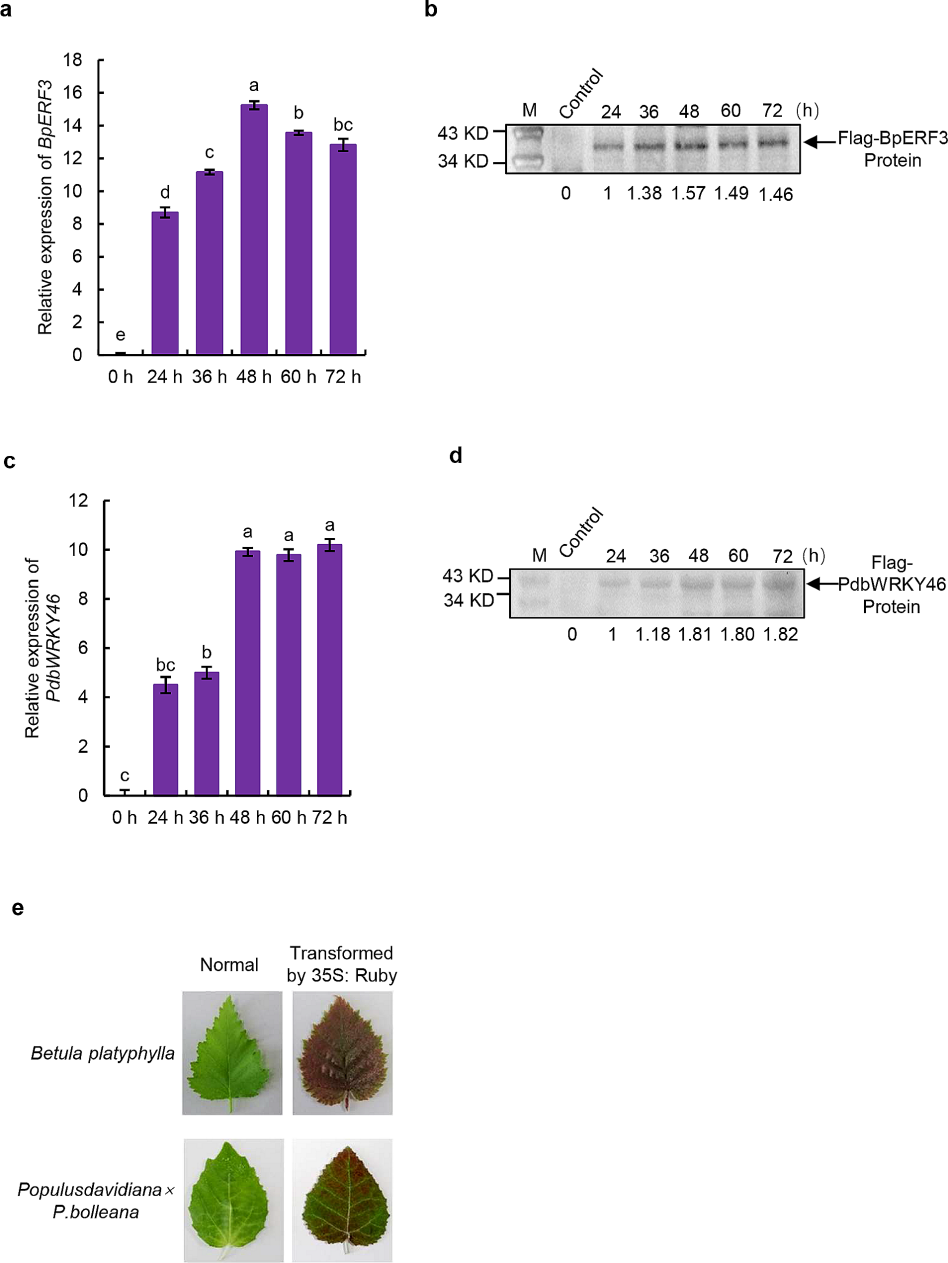
Determination of the suitable time for isolating transformed protein from transiently transformed plants (**a**) The expression of *BpERF3*-Flag in transiently transformed birch at different transformation time points. (**b**) Western blotting analysis of the BpERF3-Flag fusion protein in transiently transformed birch at different transformation time points. (**c**) The expression of *PdbWRKY46*-Flag in transiently transformed poplar at different transformation time points. (**d**) Western blotting analysis of the PdbWRKY46-Flag fusion protein in transiently transformed poplar at different transformation time points. (**e**) Distribution of the transformed cells in plants’ leaves after transient transformation for 48 h using RUBY reporter. Control: Birch plants transiently transformed with empty pCambia1307 vector. The experiment was performed three times with similar results. Error bar indicates standard deviation (SD) from the three experiments. a, b, c d and e indicate multiple comparison difference (LSD - t test, 0.05)

Previously, we confirmed that BpERF3 can bind to the promoter region of *WRKY28*, which contains a DRE cis-acting element [29]. At the same time, we also identified a WRKY protein (PdbWRKY46) from Shanxin poplar that can bind to W-boxes to regulate its target genes [31]. In the present study, we used BpERF3 from birch and PdbWRKY46 from poplar for an EMSA experiment. Both *BpERF3* and *PdbWRKY46* were fused separately with a Flag tag (*BpERF3*-Flag, *PdbWRKY*-Flag) and cloned into pROKII for overexpression. First, we generated transiently transformed plants overexpressing *BpERF3*-Flag in birch and overexpressing *PdbWRKY46*-Flag in poplar, and the expression levels of transformed *BpERF3*-Flag and *PdbWRKY46*-Flag in transiently transformed plants were determined using qRT-PCR. The expression of *BpERF3*-Flag could be detected after at 24 h after transformation, reached a peak at 48 h post-transformation, and then decreased to a still relatively high level at 72 h post-transformation (Fig. 1a). Western blotting showed that the protein level of *BpERF3*-Flag was high from 48 to 72 h post-transformation (Fig. 1b). The expression of *PdbWRKY46*-Flag could be detected at 24 h after transformation, and gradually increased, reaching a peak level at 48–72 h after transformation (Fig. 1c). Consistently, the protein maintained a high level from 48 to 72 h posttransformation (Fig. 1d). These results suggested that the transformed protein was expressed successfully, and protein could be most suitably isolated at 48–72 h post-transformation. In addition, we further studied the distribution of the transformed cells in different tissues of the transiently transformed plants by using RUBY reporter [32]. The results showed that the transformed plants can be detected nearly in the entire leaves (Fig. 1e), suggesting that most of cells had been transformed using this kind of method, and the transformed cells can express the proteins efficiently.

### Immunoprecipitation analysis

**Fig. 2:**
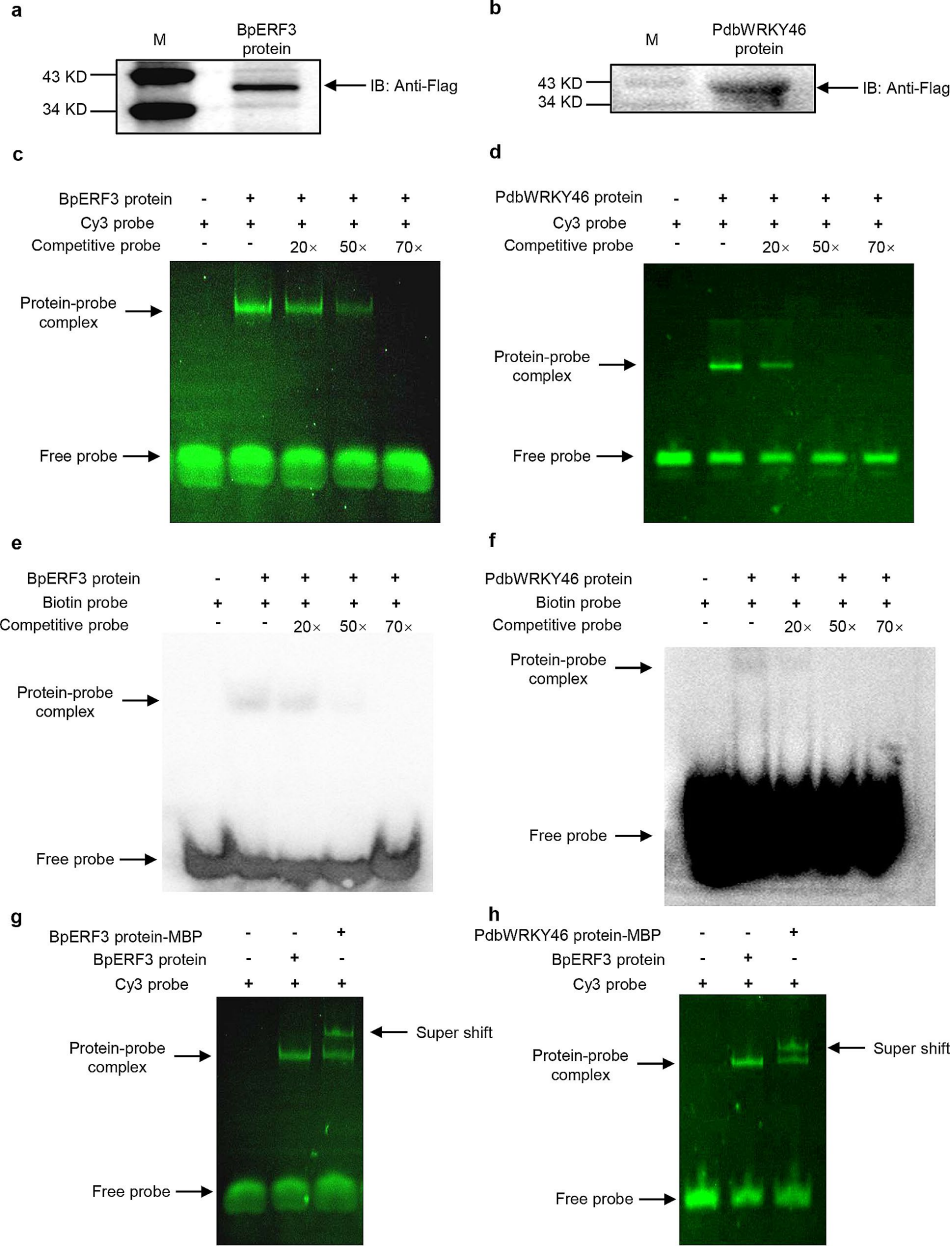
Fluorescent EMSA and super-shift fluorescent EMSA using proteins from birch and poplar plants (**a**), (**b**) Western blotting determination of the BpERF3-Flag (**a**) and PdbWRKY46-Flag (**b**) isolated from birch and poplar by immunoprecipitation. (**c**), (**d**) Fluorescent EMSA performed using the BpERF3-Flag (**c**) and PdbWRKY46-Flag (**d**). (**e**), (**f**) Chemiluminescent EMSA performed using the BpERF3-Flag (**e**) and PdbWRKY46-Flag (**f**). (**g**), (**h**) Super-shift fluorescent EMSA was performed with immune interaction using anti-Flag antibodies for BpERF3-Flag (**g**) and PdbWRKY46-Flag (**h**)

The BpERF3-Flag and PdbWRKY46-Flag proteins were isolated from the transiently transformed plants using IP with anti-Flag antibodies. The isolated BpERF3-Flag and PdbWRKY46-Flag proteins were detected using western blotting with anti-Flag antibodies. The results showed that both BpERF3-Flag and PdbWRKY46-Flag proteins had been successfully isolated (Fig. 2a, b). For BpERF3 probe labeling, the truncated promoter of *WRKY28* containing ERF binding sites (299 bp) was amplified by PCR using the primers that labeled with Cy3 at the 5’end. For PdbWRKY46 probe labeling, the paired single strand DNA containing three tandem copies of W-box was synthesized and labeled with Cy3 at the 5’ end, and annealed into double strand DNA as probe.

### Fluorescent EMSA

Fluorescent EMSA was performed and the DNA-protein complex was observed using a fluorescence imaging system. The results showed that the BpERF3 protein-DNA and PdbWRKY46 protein-DNA complexes could both be observed, and the fluorescence signal decreased when the binding solution contained an abundance of competitive probe (Fig. 2c, d). We also used chemiluminescent EMSA to study the binding of BpERF3 or PdbWRKY46 to their probes. However, the binding signals were substantially weaker than fluorescent EMSA (Fig. 2e, f). These results suggested that fluorescent EMSA can be used to detect the interaction between DNA and protein from plants, while chemiluminescent EMSA is not as sensitive under our conditions.

In addition, super-shift EMSA was usually used to detect DNA-protein complex bands that can be altered by any specific antibody [33]. We further performed super-shift fluorescent EMSA for BpERF3-Flag and PdbWRKY46-Flag protein using anti-Flag antibodies. The results indicated that in both BpERF3Flag and PdbWRKY46-Flag analysis, two retarded bands appeared, and one retarded band (one with antibody) appeared above the other retarded band (without antibody) (Fig. 2g, h). This result suggested that the quantity of proteins isolated from host plants is enough for super-shift fluorescent EMSA analysis.

### Suitable PAGE for use in super-shift fluorescent EMSA for large molecular weight DNAprotein complexes

**Fig. 3:**
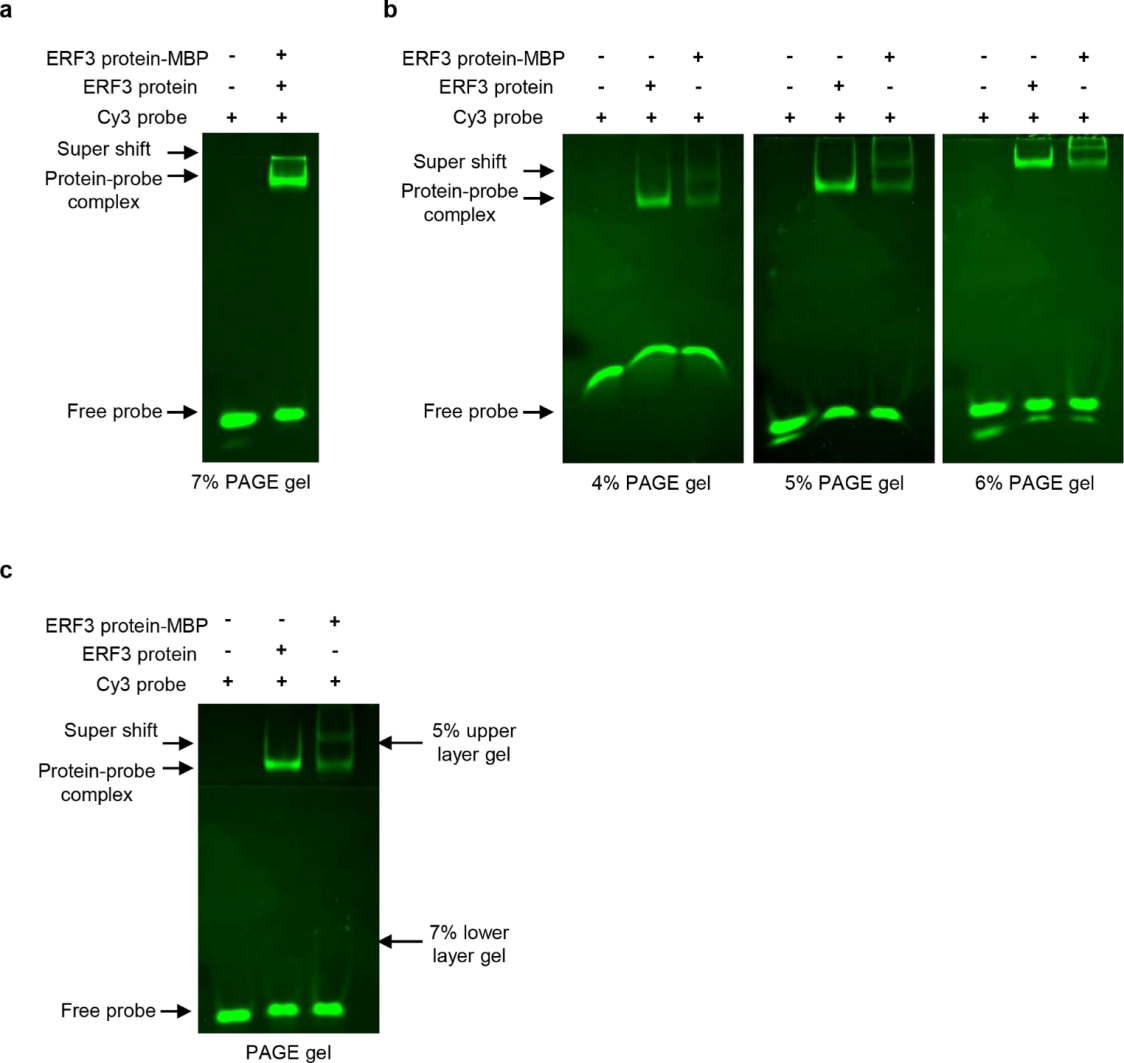
Determination of the suitable PAGE for super-shift fluorescent EMSA for large molecular weight DNA-protein complexes (**a**) The large molecular weight DNA-protein complex cannot enter a 7% PAGE gel. (**b**) 4, 5 and 6% PAGE was used in super-shift fluorescent EMSA when using large molecular weight complexes to perform EMSA. The low concentration of PAGE caused the band representing the free probe to bend. (**c**) Two concentrations of PAGE were used for super-shift fluorescent EMSA, and 5% and 7% PAGE served as the stacking and main gels, respectively

Here we also tested if the super-shift fluorescent EMSA could be used for the plant protein BpERF3-MBP expressed in *E. coli* ER2523, and explored the effects of different polyacrylamide concentrations. The complex comprising BpERF3-MBP, antiMBP antibody, and the DNA probe, is bigger than the complex of BpERF3-Flag, anti-Flag antibody, and DNA probe. When a 7% polyacrylamide gel was used for electrophoresis, the complex band representing BpERF3-MBP, anti-MBP antibody, and the DNA probe could not enter the PAGE gel, and was stuck at the top. Therefore, we used 4, 5, and 6% polyacrylamide gels for supershift fluorescent EMSA. The results showed that although the complex band (anti-MBP antibody, BpEFR3MBP, and DNA) could enter the gel to show a retarded band, the free probe displayed bent bands (Fig. 3a). In addition, when a 6% polyacrylamide gel was used, the retarded band and the super retarded band could not be resolved (Fig. 3b), suggesting that 6% PAGE is also not suitable for large molecular weight DNA-protein complexes. Therefore, we employed two concentrations of polyacrylamide, i.e. the upper (stacking) gel comprised 5% polyacrylamide to resolve the complex band, and the lower (main) gel used 7% polyacrylamide to resolve the free probe (Fig. 3c). The results showed that three kinds of neat bands were displayed in the PAGE gel, suggesting that the super-shift fluorescent EMSA was successful.

### The binding affinities of protein to DNA motifs between proteins with and without PTMs

**Fig. 4:**
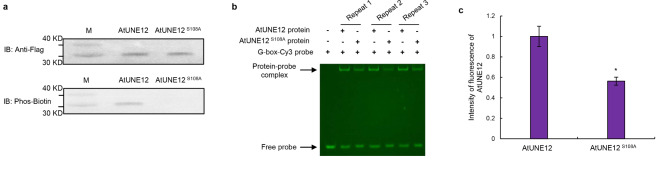
Comparison of the binding of DNA to proteins with and without phosphorylation (**a**) Determination of phosphorylation modification of AtUNE12 and AtUNE12^S108A^ proteins using western blotting with Phos™ Biotin. The quantities of AtUNE12 and AtUNE12^S108A^ proteins using western blotting with antiFlag antibody. M: protein marker. (**b**) Comparison of the binding of AtUNE12 and AtUNE12^S108A^ proteins to G-box. PPF-EMSA was performed to determine the binding of AtUNE12 and AtUNE12^S108A^ proteins to G-box. (**c**) Quantitative analysis of fluorescence intensity of PPF-EMSA. The experiment was performed three times with similar results. Error bar indicates standard deviation (SD) from the three experiments. * indicates *P* < 0.05 (t test)

As some PTMs can affect the binding of TF proteins to DNA motifs, we studied whether the fluorescent EMSA can be used to detect the binding affinities between proteins with and without PTMs. Previous study showed that AtUNE12 (a bHLH TF, Locus tag: AT4G02590) protein from Arabidopsis with phosphorylation can enhance its binding to DNA motifs [34]. In the present study, we studied whether the increase of binding affinity of AtUNE12 to DNA motifs conferred by phosphorylation modification can be detected by protein from plants fluorescent EMSA method (PPF-EMSA). The AtUNE12 proteins and AtUNE12^S108A^ protein (where Ser was mutated to Ala to abolish phosphorylation modification) were respectively isolated from Arabidopsis using IP, and quantified using Bicinchonininc Acid (BCA) method. The phosphorylation modification of AtUNE12 and AtUNE12^S108A^ was first determined using Phos-tag™ Biotin, and the results showed that AtUNE12 can be phosphorylated and AtUNE12^S108A^ cannot (Fig. 4a). At the same time, western blotting showed that these two kinds of proteins can be detected and with similar hybridization signal intensities, suggesting that the same quantity of AtUNE12 and AtUNE12^S108A^ were used (Fig. 4a). Binding of AtUNE12 and AtUNE12^S108A^ to G-box were detected using fluorescent EMSA with the same quantity of protein. The results showed that the binding affinity of AtUNE12 is substantially higher than that of AtUNE12^S108A^ when using the same quantity of AtUNE12 and AtUNE12^S108A^ (Fig. 4b), which is consistent with quantitative analysis of fluorescence intensity of EMSA (Fig. 4c). These results suggested that PPF-EMSA can be used to determine the binding affinity using both quantitative and qualitative ways.

### Determination of the sensitivity between chemiluminescent and fluorescent EMSA

**Fig. 5:**
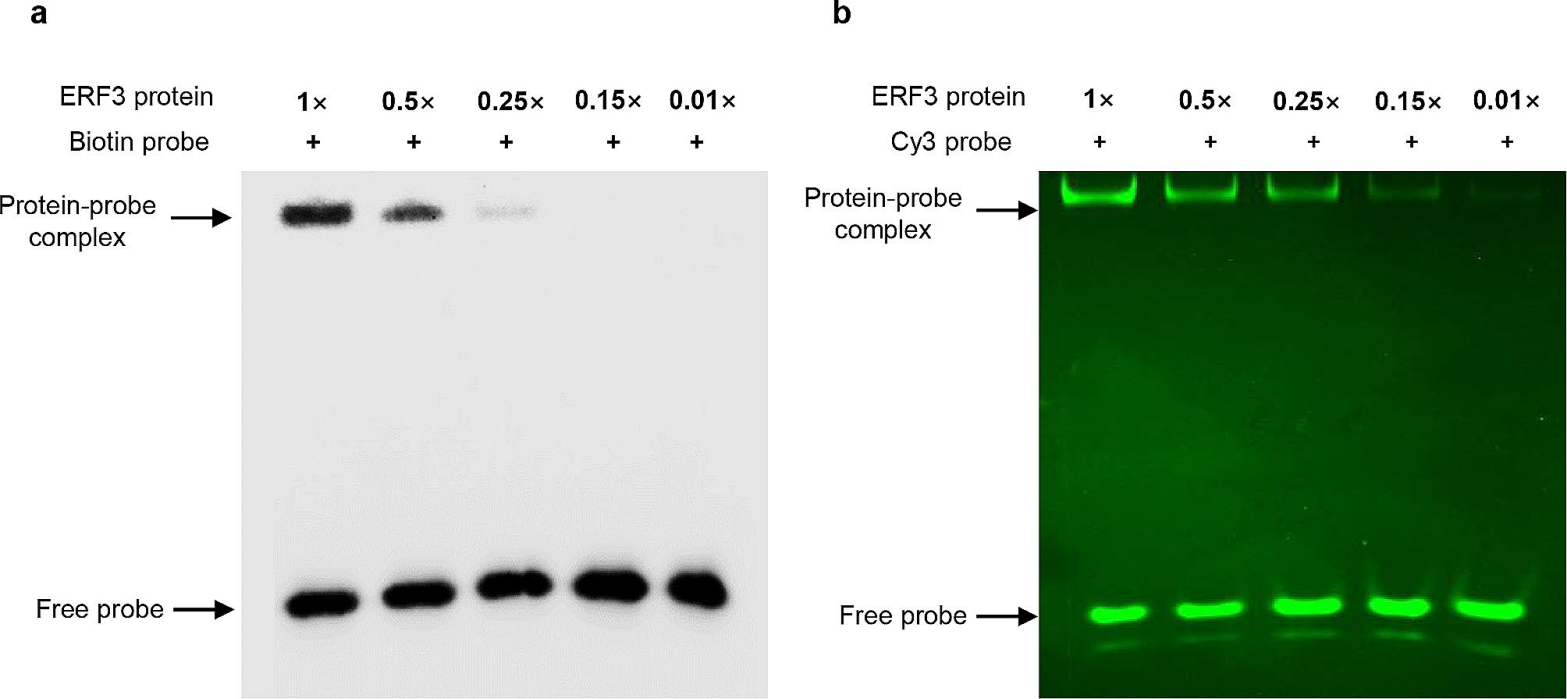
Comparison of the sensitivity between chemiluminescent EMSA and fluorescent EMSA (**a**), (**b**) The same quantity of BpERF3 protein isolated from *E. coli* strain ER2523 was used in both chemiluminescent (**a**) and fluorescent EMSA (**b**). The protein was diluted by 0.5, 0.25, 0.15 and 0.01-fold, respectively, and used for EMSA.

To compare the sensitivity between chemiluminescent and fluorescent EMSA, the BpERF3 CDS was fused with the MBP CDS, cloned into the pMAL-c5X vector, prokaryotic-expressed in *E. coli* strain ER2523, and purified using amylose resin. The purified BpERF3 protein was diluted to different concentrations (1×, 0.5×, 0.25×, 0.15×, and 0.01×). After BpERF3 protein dilution, EMSA was performed with biotin-labeled and Cy3-labeled DNA probes using the serial dilution of BpERF3-MBP, respectively. The results showed that the signal of protein and DNA in chemiluminescent EMSA became very weak when the BpERF3 protein was diluted to 0.25×, and no band was observed when BpERF3 was diluted to 0.15× (Fig. 5a). However, the DNA-protein signal in fluorescent EMSA could be observed even when the protein was diluted to 0.01× (Fig. 5b). These results suggest that the sensitivity of fluorescent EMSA was 15 times higher than that of chemiluminescent EMSA.

## Discussion

The EMSA is used to detect the interaction between DNA and protein, both qualitatively and quantitatively [15]. Until now, most proteins used in EMSA were synthesized using prokaryotic expression systems. A protein expressed using a prokaryotic expression system differs from the naturally produced eukaryotic protein in many aspects. For instance, some prokaryotic-expressed proteins cannot fold correctly, leading to decreased activity or inactivation [35], which might affect their DNA binding activity. At the same time, proteins expressed by eukaryotic organisms usually have PTMs, such as phosphorylation, acetylation, succinylation, and methylation [36]. Previous studies showed that PTMs might affect protein binding to DNA [34]. However, the proteins from host plant species can not only fold well, but also have the native PTMs. These natural states can reflect the true interaction between protein and DNA. Therefore, when studying proteins from eukaryotic organisms using EMSA, the protein expressed from host eukaryotic organism is likely to yield more accurate results than the prokaryotic-expressed version.

However, there are some limitations that restrict the use of eukaryotic proteins in EMSA. One is the sensitivity of EMSA, with chemiluminescent EMSA being unable to detect low abundance proteins (Figs. 2 and 5). Another limitation is that the target protein needs to be overexpressed in the host organism to enable its isolation using IP for two reasons. Firstly, it is difficult to isolate proteins with a low expression level, leading to difficult in isolation. Secondly, during overexpression, the target protein can be fused with an affinity tag, which is convenient for IP using antibodies of recognizing the tag. However, the generation and propagation of stable transgenic plants usually requires at least 6 months, making isolation of the protein from plant species not feasible. Therefore, the use of a transient transformation method to synthesize protein is necessary. However, most transient transformation methods for plants do not result in the foreign gene being expressed in the entire plant, which will highly reduce the production of protein. Previously, we developed a method to transiently transform genes into plants mediated by *A. tumefaciens* [37, 38], through soaking plants in *A. tumefaciens*, and the protein can be overexpressed in whole plants to improve the yield (Fig. 1e), thus facilitating the convenient isolation of the target protein. In addition, this transient transformation can be adapted all the plant species that can be transformed using *A. tumefaciens*, and does not dependent on whether a stable transformation system is available or not. At the same time, the fluorescent EMSA technology should be employed because it is at least 15 times more sensitive than chemiluminescent EMSA (Fig. 5). The results showed that PPF-EMSA that combined with fluorescent EMSA and plant transient transformation could be used successfully to determine the interaction between DNA and proteins from plants, and could also be used to perform super-shift EMSA (Fig. 2g, h). In addition, this method not only can determine the DNA bound by protein with PTM, but also can be used to compare DNA binding affinity of proteins with quantitative and qualitative ways (Fig. 4). The reason why the phosphorylated AtUNE12 binds to more DNA than unphosphorylated AtUNE12 may be that the phosphorylated AtUNE12 binds to DNA mots faster than the status without phosphorylation, causing that more DNA are bound by phosphorylated AtUNE12 in one hour. Two gel concentrations can be used to solve the problem of the DNA-Protein-protein complex being too large to be resolved using more than 6% PAGE (Fig. 3), enabling this method can detect a wide range of protein complex binding to DNA. The advantage of this method is that transient transformation is used, which can overexpress the genes in host plants quickly and efficiently, enabling that isolation of protein using IP is efficient. Although prokaryotic expression can produce the protein more efficiently, but sometimes, prokaryotic expression of proteins is difficult after transient transformation in plants for the reason that inclusion bodies form due to incorrect protein fold, which usually makes isolated protein be difficult.

DNA affinity purification sequencing (DAP-seq) is a method to discover a TF-binding sites (TFBSs), which could be combined with next-generation sequencing and affinitypurified TFs. In this technology, an affinity-tagged in vitro-expressed TF is incubated with DNA library, and the DNAs bound by TFs are isolated using the affinity tag [39]. Our method can also be used in DAP-seq to prepare affinity-tagged TFs from host plants, which might be helpful to discover TFBSs. In addition, PPF-EMSA method could be used to determine whether the PTMs of protein can affect their DNA binding capability, such as the present studies (Fig. 4). As fluorescence is easy to quantify, PPF-EMSA could be used for comparing the bindings of a natural protein to different DNA motifs by quantitating fluorescence intensities, which might be more reliable than the use of the proteins expressed from prokaryotic expression system. Therefore, this system might have a wide application in studies of the interactions between DNA and protein, and protein-protein and DNA.

### Troubleshooting

Fluorescent EMSA usually does not suffer from high background noise, or speckling/spots because the results can be observed directly on the PAGE gel, and does not need membrane transfer and washing, equilibration, and chemiluminescent substrate treatment. However, some issues might still arise in PPF-EMSA. These are listed, with their causes and solutions, in Table 1, which might be helpful in performing fluorescent EMSA smoothly.

**Table 1:** Problem, causes, and solutions associated with PPF-EMSA.

Problem	Cause	Solution
No retarded band but has a free probe band	Low concentration of protein	Concentrate the protein using a protein ultrafiltration column with an appropriate cutoff.
	Low quantity of protein caused by low efficiency of transient transformation	(1) Optimize the transient transformation method, and determine the suitable time for protein isolation using western blotting. (2) Use more transiently transformed material.
	The quality or purity of the fluorescent probe is poor	When probe is more than 200 bp long, purify the probe using chloroform extraction. After extraction, add 3-times the volume of ethanol and glycogen to precipitate the probe. When probe is less than 200 bp long, purify the probe using chloroform extraction twice, and the supernatant can be used for EMSA directly.
	The two single DNA strand probes do not anneal	Denature the probes in 1× TE buffer at 98 °C for 2 min, and leave them at room temperature for 30 min to anneal.
Retarded band at the top of gel	The concentration of the gel is too high	Reduce the concentration of the gel or use a low concentration gel as an upper gel and a high concentration gel as lower gel.
More retarded bands appear than expected	Additional bands are usually caused by non-specific binding	The protein needs to be purified or the amount of protein used should be reduced.
No bands detected/low signal	The target DNA is not fluorescently labeled or not fluorescently labeled enough	Use target DNA with end-labeled fluorescence.
	Insufficient labelled target DNA was used	Increase the target DNA concentration.
	The target DNA was degraded	Check the integrity of the target DNA.
Irregular DNA-protein band or free probe band	High temperature during PAGE gel electrophoresis	Reduce the voltage to decrease the temperature of PAGE or carry out PAGE in an ice batch.
	The concentration of the gel is too low	Adjust the concentration of the gel.

## Conclusion

In this study, we developed a technique to quickly isolate proteins of interest from host plants and then the isolated proteins can be used in EMSA, especially suitable for fluorescent EMSA, which termed PPF-EMSA method. A special transient transformation method was employed that can transiently deliver transgenes into the plant, enabling efficient synthesis the proteins encoded by transgene. Then, the target protein was isolated using immunoprecipitation and used for fluorescent EMSA and fluorescent super-shift EMSA. This method not only can determine the protein binding to DNA, but also can detect the effect of protein DNA interaction affected by post translationally modification. The PPF-EMSA system provides an approach for determining protein and DNA binding in a natural state, and also as convenient as use of protein from prokaryotic expression system. This technology system can adapt to all the plant species being transformed by *A. tumefaciens*, and not depending on whether stable transformation has or not, and therefore it will have a wide application.

### Electronic supplementary material

Below is the link to the electronic supplementary material.

Supplementary Material 1

## Data Availability

Data is provided within the manuscript or supplementary information files.
