# Supplementary material for: An electrophoretic mobility shift assay using the protein isolated from host plants
Source: Plant Methods. 2024 May 12;20:68. doi: 10.1186/s13007-024-01201-7 (PMC11089672; doi:10.1186/s13007-024-01201-7)
Supplement: Supplementary file 1 — Supplementary Material 1 [file 13007_2024_1201_MOESM1_ESM.docx]

**Supplementary Table 1 The primers used for constructing the prokaryotic expression vectors.**

The primers used in construction of the prokaryotic expression vectors

| Gene names | Primer sequences (5’-3’);  F: Forward; R: Reverse |
| --- | --- |
| C5x-BpERF3 | F: GGAATTCCATATGATGTGTATGCCAAAGGCAAAGG  R: CGCGGATCCCTACCTAGTGGGAGGAGTATG |
| C5x-PdbWRKY46 | F: CGCGGATCCATGGAATTGTCCATGGAATG  R: GGAATTCCTAAGCAAAGAAATCTGGG |

**Supplementary Table 2 The primers used for constructing plant expression vectors.**

Primer sequences for vector construction

| Gene names | Primer sequences (5’-3’);  F: Forward; R: Reverse |
| --- | --- |
| P1307-BpERF3 | F: CGAGCTCATGTGTATGCCAAAGGCAAAGG  R: GCTCTAGACTACCTAGTGGGAGGAGTATG |
| P1307-PdbWRKY46 | F: GCTCTAGAATGGAATTGTCCATGGAATG  R: CGGGGTACCCTAAGCAAAGAAATCTGGG |

**Supplementary Table 3 The primers used for amplifying DNA probes of EMSA.**

Primer sequences for EMSA DNA probes

| Gene names | Primer sequences (5’-3’);  F: Forward; R: Reverse |
| --- | --- |
| EMSA-WRKY28 | F: ATACATATACTTACCTCTTC (5’Cy3)  R: ATCGGCAGCGTTTCATATGG |
| EMSA-WRKY28 | F: ATACATATACTTACCTCTTC (5’biotin)  R: ATCGGCAGCGTTTCATATGG |
| EMSA-W-box | F: TTGACTTGACTTGAC (5’Cy3)  R: GTCAAGTCAAGTCAA |
| EMSA-W-box | F: TTGACTTGACTTGAC (5’biotin)  R: GTCAAGTCAAGTCAA |

**Supplementary Table 4 The primers used for qRT-PCR.**

Primer sequences for qRT-PCR

| Gene names | Primer sequences (5’-3’);  F: Forward; R: Reverse |
| --- | --- |
| Tubulin | F: TCAACCGCCTTGTCTCTCAGG  R: GGTTGTCACGTAAGCTCGGT |
| RT-qERF3 | F: AGACAGCTGCAGAACCAAG  R: GGGAAGTTGAGTTTGGCC |
| Actin | F: GCTGAGAGATTCCGTTGCCCTG  R: GGCGGTGATCTCCTTGCTCATT |
| RT-qWRKY46 | F: CATGTAACCAAGCCTCTCC  R: CCTTAGAGCCCAAGTCCTC |
